# So you think you can dance? Development and validation of the Dance Self-Efficacy Scale for older adults (DanSES-60+) for research and practice

**DOI:** 10.3389/fpsyg.2025.1712057

**Published:** 2026-01-07

**Authors:** Martha Waugh, Rhiannon Lee White, Celia B. Harris, Dafna Merom

**Affiliations:** 1 School of Health Sciences, Western Sydney University, Penrith, NSW, Australia; 2 The MARCS Institute for Brain, Behaviour and Development, Western Sydney University, Sydney, NSW, Australia; 3 Translational Health Research Institute, Western Sydney University, Sydney, NSW, Australia

**Keywords:** dance and movement, health, exercise, self-efficacy, older adults, scale development, individual differences, evaluation

## Abstract

**Background:**

Dance and movement represent a recommended form of exercise for older adults that benefits health across diverse cultures and socioeconomic groups. Individual differences in dance self-efficacy may be key determinants of participation in dance for health programs, yet existing measures for assessing these psychological factors in older adults are limited. Scale development research addressing this gap could enhance understanding of factors influencing engagement and program success in dance-based health interventions. This study developed and validated the Dance Self-Efficacy Scale for older adults (DanSES-60+).

**Methods:**

The existing 6-item dance self-efficacy measure was extended through literature review and focus group analysis. Expert review reduced 60 items to 32 items, which were administered via survey to 289 community-dwelling older adults (*M* age = 72.1 years). The sample was stratified and split for exploratory (*n* = 97) and confirmatory (*n* = 192) factor analysis. Scale reliability and validity were assessed following established psychometric scale development procedures, including tests of internal consistency, test-retest reliability (2-week interval; *n* = 80), and associations with relevant constructs and demographic factors.

**Results:**

Item analysis reduced the scale to 12 items with a theoretically meaningful two-factor structure: Barriers and scheduling self-efficacy (confidence in overcoming attendance and participation challenges) and dance task self-efficacy (confidence in performing dance activities). The DanSES-60+ demonstrated excellent internal consistency (α = 0.95) and good test-retest reliability (ICC = 0.84), with strong criterion and construct validity evidence. Dance self-efficacy scores were significantly positively associated with prior dance experience, current dance participation, female gender, mobility, general health, physical activity levels, and engagement in arts and creative groups. Age, falls history, education level, and cultural and/or linguistic diversity showed no relationship with scores.

**Discussion:**

The DanSES-60+ addresses a critical measurement gap in dance and aging research, providing the first psychometrically robust tool for assessing dance self-efficacy in older adults. The scale enables participant screening and stratification, program evaluation, progress monitoring, and investigation of psychological mechanisms underlying dance program effectiveness. Established scale cutoffs and confidence indicators enhance practical utility for applications in dance for health research and practice.

## 1 Introduction

Dance offers a promising, safe, cost-effective and widely accessible approach to promote healthy, active ageing in community settings (Fancourt et al., 2020; Izquierdo et al., 2021; Waugh et al., 2024). The integration of shared, embodied arts experiences and aerobic and anaerobic exercise allows dance to simultaneously address multiple aspects of wellbeing in aging (Izquierdo et al., 2021; Clifford et al., 2023). However, our recent scoping review (Waugh et al., 2024) demonstrated that adherence to dance programs over 3 months is similar to adherence rates for other exercise programs (Picorelli et al., 2014; Farrance et al., 2016). These findings challenge assumptions that dance, as a social, mind-body activity with music and expression, is both accessible and inherently more enjoyable and engaging than standard exercise options for older people and would therefore promote greater adherence (McCrary et al., 2021; Haynes et al., 2023). Combined with inconsistent outcomes across studies evaluating the health benefits of dancing in later life (McCrary et al., 2021; Clifford et al., 2023; Lazo Green et al., 2024), the collective evidence indicates a need to investigate factors determining program success.

Dance is a complex motor skill that requires coordination, rhythm, memory, balance, physical expression, and social interaction (Kraft et al., 2015; Hackney et al., 2024; Waugh et al., 2024). These simultaneous demand**s** present many potential impediments to success, particularly for novices and older people with physical and cognitive limitations (Picorelli et al., 2014). Understanding personal factors that influence participation in complex activities is essential to support engagement and inform arts-based health initiatives (Picorelli et al., 2014; Beck et al., 2016; Carey et al., 2019; Sonke, 2021).

Self-efficacy—situation-specific confidence—is a central construct in Bandura’s Social Cognitive Theory (SCT) and a pivotal determinant of health behaviors in later life (Bandura, 1997, 1998, 2012; McAuley et al., 2011b). Self-efficacy beliefs influence activity choices, effort expenditure, perseverance, and emotional responses to challenges (Maddux, 1995; Bandura, 1998, 2012). Research has established the role of self-efficacy in predicting exercise uptake and participation among older people, and the positive association between self-efficacy and exercise behavior, functional mobility, mental health, and cognitive abilities (Van Stralen et al., 2009; McAuley et al., 2011b). Self-efficacy may therefore be a key determinant for participation in group arts activities requiring multiple capabilities.

Exercise interventions for older people often incorporate strategies to enhance activity-specific self-efficacy including peer support, goal setting, graded tasks, and self-monitoring (Chase, 2015; Lachman et al., 2018; Carey et al., 2019; Gilchrist et al., 2024). However, these theory-informed approaches to support exercise adherence and engagement are underutilized in dance. Our scoping review of 116 dance programs for older adults found only one initiative—BALIAMOS Latin dance program in low-income Latino communities (Marquez et al., 2021)—was explicitly SCT-informed, incorporating strategies designed to provide health education and social support, and boost exercise self-efficacy (Waugh et al., 2024). Given dance’s potential as a health resource, particularly for communities with health disparities and barriers to physical activity (Fancourt et al., 2020; Golden et al., 2023; Waugh et al., 2024), incorporating theory-informed approaches in dance programs represents a missed opportunity to enhance program effectiveness. A comprehensive measure of dance self-efficacy would be valuable for participant screening, progress monitoring, program evaluation, and to examine mechanisms underlying program effectiveness.

Dance self-efficacy is defined as a person’s beliefs in their capabilities to participate in organized group dance. Initial investigations used a 6-item dance self-efficacy measure developed for two large-scale for older people investigating the effects of group dance on cognitive health (Merom et al., 2016a) and for falls prevention (Merom et al., 2016b). Our secondary analysis of data from these trials found that stronger dance self-efficacy at baseline was associated with more past dance experience, better mental health, more frequent exercise, better physical and cognitive function, and more social support, factors likely to influence capability for group dancing (Waugh et al., 2023). Higher attendance at the dance program improved dance self-efficacy, while low attenders showed decreased dance self-efficacy.

We conducted a further study to examine the associations between dance self-efficacy, program participation and satisfaction, and health outcomes, using data from the falls prevention study only (Merom et al., 2016b; Waugh et al., 2025). Improved physical function (using the Short Physiological Performance Battery; Guralnik et al., 1994) occurred only in participants with lower baseline dance self-efficacy and physical function improvements were explained (fully mediated) by gains in dance self-efficacy. Both high program attendance and increased dance self-efficacy were associated with greater program satisfaction. Results suggest both that the program was only effective to improve physical function for people with low dance self-efficacy at baseline and a mechanistic link between improved dance self-efficacy and improved physical function and adherence.

Self-efficacy is an established determinant of both exercise and arts engagement (Van Stralen et al., 2009; Picorelli et al., 2014; Sonke, 2021), making reliable measurement tools essential. Although the preliminary 6-item dance self-efficacy measure provided valuable insights, it had substantial limitations that needed to be addressed. The 6-item measure focused on executing dance tasks, sustaining physical effort, and navigating the social environment. However, the scale had limited expert review, was not validated prior to use in the research trials, did not capture self-efficacy for maintaining participation in organized dance activities, and included two highly correlated items (Waugh et al., 2023). Additionally, the sample was restricted to participants living in Greater Sydney who enrolled in the dance research trials which somewhat limits the generalizability of the scale for use in broader older adult populations. Together, the promising initial findings and the limitations of the brief 6-item scale highlight the need for a more comprehensive and rigorously validated measure of dance self-efficacy for older adults.

General exercise self-efficacy measures (e.g., Bandura, 2006; Rodgers et al., 2008) focus on maintaining a regular exercise routine and generalized barriers to exercise (poor weather, fatigue, stress). The tools do not capture the cognitive-motor tasks and social challenges associated with group dance, or self-efficacy for scheduling organized group activities. Bandura maintained that activity-specific self-efficacy measures provide stronger predictive value than general measures (Bandura, 2006). Given the complex qualities of dance that contribute to health and wellbeing benefits across multiple domains (Waugh et al., 2024), a dance-specific self-efficacy measure is warranted.

A dance self-efficacy measure should address three domains established in exercise self-efficacy research: Task self-efficacy (confidence to perform key activity tasks); barriers self-efficacy (confidence to overcome challenges related to the activity); and scheduling self-efficacy (ability to maintain regular exercise) (Maddux, 1995; Rodgers and Sullivan, 2001; Rodgers et al., 2008). In exercise research, these three domains have been shown to predict exercise adoption, adherence, and maintenance over time, with each domain influencing different aspects of the exercise behavior chain (McAuley and Blissmer, 2000; Rodgers and Sullivan, 2001; Rodgers et al., 2008). The model is particularly relevant for dance because it captures cognitive-motor demands and the social, psychological, the practical challenges of scheduling group activities, and the shared challenges with exercise. However, group dance may involve additional dimensions such as creativity, rhythm, and performance, and therefore it remains uncertain whether the three-domain model will apply to all aspects of dance participation.

A dance self-efficacy scale for older people has many potential applications. For researchers, it enables examination of psychological mechanisms and moderators of program effectiveness (Fancourt et al., 2021). For practitioners, it can be used for participant screening and streaming, to identify participants needing additional support or modified instruction, and for monitoring progress (Waugh et al., 2023, 2025). The tool may also guide dance program design, and evaluation.

The purpose of this study was to develop and validate a comprehensive dance self-efficacy measure for community-dwelling older adults. This work extends the existing 6-item dance self-efficacy measure for older people across the three domains of exercise self-efficacy. The revised scale will include scheduling self-efficacy for organized group dance activities and investigates self-efficacy for dance tasks and barriers to participation across various dance programs and styles (Waugh et al., 2024). Our approach builds upon qualitative and quantitative work exploring dance self-efficacy in older age (Sonke, 2021; Waugh, 2022; Waugh et al., 2023, 2025) and our scoping review of the design and delivery of 116 dance for health programs for older people (Waugh et al., 2024). We aimed to address the limitations of the original scale while maintaining its demonstrated utility.

## 2 Materials and methods

### 2.1 Design and procedure

Ethical approval for the development of the Dance Self-Efficacy Scale for older adults (DanSES-60+) was obtained from Western Sydney University (Ethics Approval: H16210; DAnCE for falls prevention trial, Ethics Approval: H9468). We followed systematic scale development procedures and reporting recommendations (Bandura, 2006; DeVellis, 2017; Boateng et al., 2018; Carpenter, 2018). The 6-stage development process is outlined in Table 1.

**TABLE 1:** Stages of development of the DanSES-60+.

Stage	Procedure
1.	Domain identification and item generation a. Literature review of age-related factors likely to impact dancing and relevant psychometric instruments b. Deductive qualitative analysis of focus group data from older adult dance program participants c. 60 items generated (54 new + 6 original items)
2.	Content validity assessment a. Items grouped into three subscales: Dance tasks, barriers, and scheduling self-efficacy b. Expert review by nine specialists c. Items refined based on expert reviewer feedback, pool reduced to 32 items
3.	Survey administration a. 32 dance self-efficacy items administered to sample (*N* = 289) b. Stratified randomization into Sample 1 (*n* = 97) and Sample 2 (*n* = 192)
4.	Item analysis and reduction a. Item-level statistics, inter-item and item-total correlations examined b. Seven redundant items removed, 25 items retained for factor analysis
5.	Factor analysis and scale construction a. Exploratory factor analysis (EFA) conducted on original six items and 25-item set using Sample 1 b. Strategic item selection based on factor structure and theoretical coverage c. Final 12-item, two-factor scale validated with confirmatory factor analysis (CFA) on Sample 2
6.	Item analysis of revised scale (12 items; two subscales) a. Internal reliability of subscales after item removal b. Calculation of scale scores c. Tests of temporal stability (test-retest reliability over 2 weeks; *N* = 80) d. Tests of criterion and construct validity and assessment of confidence indicators

### 2.2 Scale development and item generation

Development of the DanSES-60+ as informed by substantive literature reviews examining self-efficacy theory (Maddux, 1995; Bandura, 1997, 1998, 2012), and age-related factors likely to impact dancing and participation in group activities. Content domains were guided by reviews of exercise self-efficacy (Resnick and Jenkins, 2000; Bandura, 2006; Rodgers et al., 2008; McAuley et al., 2011b), motor self-efficacy (Potter et al., 2009), falls self-efficacy (Yardley et al., 2005), and creative self-efficacy (Karwowski et al., 2018).

To ensure content validity, we generated items through deductive, theory-driven thematic analysis of focus group data from the DAnCE cluster-randomized control trial (*N* = 10 focus groups, 60 participants; Merom et al., 2016b). Our analysis identified specific dance tasks participants found challenging and barriers to participation. We incorporated insights from qualitative studies exploring older adults’ dance experiences (e.g., Ali-Haapala et al., 2020; Haynes et al., 2023) and features of dance programs identified in our scoping review (Waugh et al., 2024).

From these sources, we identified key dance self-efficacy concepts, organized under three exercise-related self-efficacy dimensions:

1. Task self-efficacy: Performing dance moves and choreographed sequences; cognitive tasks of group dance; engagement with artistic and creative tasks; and endurance for dancing.
2. Barriers self-efficacy: Dancing in social contexts; psychological barriers to enjoying dancing (being watched, poor performance); and personal barriers to dance (fatigue, stress).
3. Scheduling self-efficacy: Maintaining regular attendance at organized group dance activities in general, and despite competing demands on time.

Item content directly referenced older adults’ descriptions of their dance experiences. Concept and item development is further outlined in Waugh (2022). The initial pool of 60 items included the original six items from previous research, with two items rephrased for clarity. Original items assessed dancing in time with music, remembering steps, following instructions, enjoying dancing with a group, dancing with unknown partners, and coping with the physical effort involved in dancing, and covered task and barriers self-efficacy. The 54 new items covered all aspects identified through the above literature review and qualitative research on older people’s dance engagement.

### 2.3 Content validity assessment

Nine expert academic researchers, health professionals, and dance health practitioners reviewed the 60-item pool. The expert reviewers were recruited from professional networks and represented diverse fields of expertise. This interdisciplinary panel included dance facilitators with over 10 years teaching experience working with clinical and non-clinical groups of older people, as well as academic researchers with expertise in dance, gerontology, exercise science, motivation and self-efficacy in exercise, community care, falls prevention, and psychometric scale development. Many had dual roles spanning both practice and research, bringing complementary perspectives to the evaluation process. Each reviewer received a package that described the DanSES-60+ purpose, brief literature review, explanation of dance self-efficacy concepts, and content validity assessment instructions. Following established recommendations (DeVellis, 2017; Elangovan and Sundaravel, 2021), experts were asked to rate each item as “essential,” “modify,” or “remove” (scored as 3, 2, 1).

Consensus between raters was fairly low due to diverse perspectives and knowledge bases regarding both self-efficacy and psychometric tools. Rather than establishing a clear inflection point, we removed the lowest scoring items from each dance self-efficacy concept to ensure breadth of representation. For retained items, we incorporated expert feedback through five modifications: simplified instructions, standalone “I can” statements, removal of ambiguous phrases, positive language throughout, and adaptation to represent varied dance styles. This reduced the item pool to 32 items.

### 2.4 Response format and survey administration

The revised 32 dance self-efficacy items were administered via an online survey. We surveyed Australian residents aged 60 and over. We defined older adults as individuals aged 60+ years to align with both research practices and community dance programs that often include participants aged under 65, as evidenced by our scoping review (Waugh et al., 2024). To ensure respondents understood questions applied to all people aged over 60 regardless of prior dance experience, the survey began: *Whether or not you have danced before, how confident are you that you can do the following*. Based on recommendations (Bandura, 2006), we used a five-point Likert scale; 1 = Not at all confident to 5 = Very confident. All items were worded positively (DeVellis, 2017) and presented in randomized order within 10–11 item blocks.

### 2.5 Measures

#### 2.5.1 Demographics

Age was assessed in years. Gender was assessed with the Australian Bureau of Statistics (ABS) item with categories Male, Female, Non-binary, Prefer different term (Australian Bureau of Statistics, 2022). Highest level of education was assessed at three-levels: Did not complete high school; Completed high school, vocational training, apprenticeship, or college (no degree); Bachelor’s degree or higher (Merom et al., 2016b). Cultural diversity was assessed with the ABS items “Were you born in Australia?” and linguistic diversity, “Do you speak a language other than English at home?” (Australian Bureau of Statistics, 2022).

#### 2.5.2 Dance experience and participation

Four items assessed dance experience. Three binary (yes/no) items from Merom et al. (2016a,b) assessed current participation (weekly, most weeks), regular participation over 5 years (weekly), and dance participation when younger (school years, 18–30; 30–45; 45+ years). Overall dance experience level was categorized using an item from the Dance Sophistication Index (GOLD-DSI; Rose et al., 2020) with five levels: None at all, Beginner, Intermediate, Advanced, and Professional.

#### 2.5.3 Health and mobility

Mobility limitations were assessed with: “Do you have any difficulty climbing a flight of stairs or walking two blocks without any help?” (Yes/No). Falls history was assessed with: “During the past 12 months, have you fallen accidentally and landed on the floor or ground, or fallen and hit an object like a table or chair?” (Yes/No). These items were used in previous dance research (Franco et al., 2020). Self-rated health used the standard item “In general, would you say your health is: Poor; Fair; Good; Very good; Excellent.” Number of chronic health conditions were measured with the Self-administered Co-morbidity Questionnaire (SCQ), with a clinically meaningful cutoff of 0–2 vs. 3+ conditions (Sangha et al., 2003).

#### 2.5.4 Physical activity and arts engagement

Self-reported physical activity of 30 mins + per day/week identified participants meeting guidelines (3 + days/week) versus not (Bauman and Richards, 2022). Participation in arts (active and receptive) and creative groups was assessed using items adapted from the 2021 U.S. General Social Survey and 2020 U.S. Arts Basic Survey (Bone et al., 2021). Items assessed (past 12 months): participation in a creative group such as a hobby group, garden club, book club, cultural group, or a study or discussion group, live and visual arts event attendance, and active participation in creative live arts (making arts or craft objects, performing). The items were summed for a total arts and creative group participation score out of five.

### 2.6 Recruitment and participant characteristics

Australian residents were recruited through the MARCS AgeLab database, Senior Centers, and dance program providers (RIPE dance, Dance for Parkinson’s Australia, Gray Panthers, Lifespan Dance, Queensland Ballet for Seniors, ZEST Dance, Ballroom Fit, line dancing organizations). The study aimed to develop a scale representative of community-dwelling older people who might participate in group dance sessions, so we placed no restrictions on mobility, cognitive ability, or dance experience. Participants provided informed consent by completing the survey.

The sample comprised 289 respondents aged 60–92 years (*M* = 72.1, SD = 6.8), 21.1% male. Using stratified randomization based on dance experience level and age, the sample was split into Sample 1 (*N* = 97; *M* age = 72.0; SD = 7.0) for scale construction and Sample 2 (*N* = 192; *M* age = 72.1; SD = 6.7) for validation analysis. Table 2 summarizes participant characteristics. All 32 dance self-efficacy items administered to the sample, along with other survey measures and participant response data, are available in the deidentified open access dataset (Waugh and Merom, 2025).

**TABLE 2:** Participant characteristics for the total sample and split samples.

Respondent characteristics	*N* total	% *N* total	Mean age (years)	% *N* sample 1	% *N* sample 2
Total	289	100.0	72.1	97	192
**Age**					
60–69 years	113	39.1	–	39.2	39.1
70–79 years	128	44.3	–	44.3	44.3
80–89 years	38	13.1	–	13.4	13.0
90+ years	3	1.0	–	2.1	1.0
**Gender**					
Male	61	21.1	71.3	19.6	21.9
Female	220	76.1	72.3	78.4	75.0
**Born in Australia**					
Yes	192	66.4	71.7	62.9	68.2
No	90	31.1	72.8	35.1	29.2
Aboriginal or Torres Strait Islander (yes)	6	2.1	67.5	0.0	3.3
**Language other than English at home**					
Yes	45	15.6	70.0	17.5	14.6
No	231	81.7	72.5	79.4	82.8
**Highest education**					
Did not complete high school	10	3.5	78.3	3.1	3.6
High school, training, or college	106	36.7	72.4	33.0	38.5
Bachelor’s degree or higher	166	57.4	71.6	61.9	55.2
**Resides**					
In the community	255	88.2	71.5	87.6	88.5
Senior independent living	26	9.0	77.7	10.3	8.3
**Difficulty climbing stairs or walking^1^**					
Yes	58	20.1	75.1	21.6	19.3
No	224	77.5	71.3	76.3	78.1
**Fall/s (previous 12 months)^2^**					
Yes	66	22.8	71.8	19.6	24.5
No	216	74.7	72.2	78.4	72.9
**Chronic health conditions^3^**					
0–2	171	59.2	72.0	52.6	62.5
3+	75	26.0	73.8	27.8	25.0
**Physically active 30 mins + days/week^4^**					
<3 days (insufficiently active)	95	32.9	71.9	33.0	32.8
3+ days (meeting PA guidelines)	187	64.7	72.2	64.9	64.6
**Self-reported dance experience level**					
None at all	28	9.7	70.0	9.3	9.9
Beginner	106	36.7	72.6	37.1	36.5
Intermediate	115	39.8	72.3	39.2	40.1
Advanced	28	9.7	71.7	9.3	9.9
Professional	10	3.5	70.9	4.1	3.1

^1^Self-reported difficulty climbing a flight of stairs and/or walking two blocks.

^2^Self-reported falls history (12 months).

^3^Number of chronic health conditions on Self-administered Co-morbidity Questionnaire (SCQ).

^4^Self-reported physical activity of 30 mins + per day/week.

### 2.7 Overview of statistical analysis

Scale development follows a systematic process to ensure the instrument measures what it intends to measure (dance self-efficacy) in the target population (people aged 60+ years) and does so reliably and consistently. Our analysis addressed five key questions: (1). Do the individual items function well? (2). What underlying factors or dimensions exist in the data? (3). Does the proposed factor structure hold up in independent samples? (4). Do the items work together as a cohesive set and do they measure dance self-efficacy reliably over time? and (5). Does the scale relate to factors we expect may affect dance self-efficacy, such as dance experience and mobility? To answer these questions, we conducted a series of statistical analyses outlined below, following established scale development and reporting guidelines (DeVellis, 2017; Boateng et al., 2018; Carpenter, 2018).

All analyses were conducted using JASP version 0.19.3. Sample size followed scale development recommendations of 200–300 participants for factor analysis, providing approximately nine participants per item in the initial pool (Clark and Watson, 1995; DeVellis, 2017).

#### 2.7.1 Item analysis and reduction

This step evaluated how individual items performed to identify and remove problem or redundant questions. Descriptive statistics and missing data patterns were examined for all items. Item distributions were assessed for normality, floor and ceiling effects, and response range. Inter-item and corrected item-total correlations and were examined to identify redundant or poorly performing items prior to factor analysis (Clark and Watson, 1995; DeVellis, 2017). Item analysis used conservative criteria: item-rest correlations below 0.30, extreme means (<1.5 or >4.5), low variance (SD < 0.75), and high missing data rates (>5%) (Streiner et al., 2015; DeVellis, 2017).

#### 2.7.2 Factor analysis

Factor analysis identified patterns in how items related to each other (clustered together) to suggest a potential underlying structure for measuring dance self-efficacy. Exploratory factor analysis (EFA) used maximum likelihood estimation with oblique oblimin rotation to allow for correlated factors, as recommended for psychological constructs (Costello and Osborne, 2005). Confirmatory factor analysis (CFA) was conducted on an independent validation sample (Sample 2) to test the factor structures identified in EFA (Boateng et al., 2018).

Model fit was assessed using multiple indices: chi-square test (χ^2^), comparative fit index (CFI), Tucker-Lewis index (TLI), root mean square error of approximation (RMSEA), and standardized root mean square residual (SRMR) (Marsh et al., 2004). CFI values ≥ 0.90 and RMSEA values ≤ 0.08–0.10 were considered acceptable fit for this psychological construct (Marsh et al., 2004). Internal consistency reliability (the degree to which scale items measure a single construct) was evaluated using Cronbach’s alpha (α) and McDonald’s omega (ω), with omega preferred for multifactor models (DeVellis, 2017). Factor correlations were assessed to determine relationships between latent constructs.

#### 2.7.3 Scale construction

This stage focused on selecting the optimal combination of items, strategically balancing theoretical coverage and construct validity alongside psychometric properties (Boateng et al., 2018). Items were retained based on factor loadings, theoretical importance, and item-total correlations (Clark and Watson, 1995; DeVellis, 2017). The selection process built incrementally upon the original 6-item scale, with items added and removed based on their contribution to construct coverage and model fit.

#### 2.7.4 Analysis of the final scale: internal reliability and scale scores

After finalizing item selection, we examined the complete scale’s performance characteristics and established guidelines for interpretation. The EFA (Sample 1) and CFA (Sample 2) samples were combined. Internal consistency reliability was evaluated for the full scale and subscales using Cronbach’s alpha (α) and McDonald’s omega (ω) (DeVellis, 2017). Total and subscale scores were calculated by summing the item responses. Descriptive statistics including means, standard deviations, ranges, and distribution characteristics were computed for final scale scores and items. Scale cutoffs for low, moderate and high dance self-efficacy were established using either quartile (25th and 75th) or tertile (33.3rd and 66.6th) percentile ranks depending on the distribution of the total scale scores.

#### 2.7.5 Temporal stability

Test-retest reliability (temporal stability) was assessed over a 2-week interval to determine if the scale produces consistent results at different time points. Pearson correlations and intraclass correlation coefficients (ICC) (2,1) with two-way random effects and absolute agreement were calculated between Time 1 and Time 2 scores. Reliability coefficients ≥ 0.70 were considered acceptable and ≥0.80 indicating good reliability (DeVellis, 2017).

#### 2.7.6 Criterion and construct validity

Validity analyses examined specific relationships between the dance self-efficacy scale and external measures to provide evidence that the instrument measures the intended construct. Criterion validity (whether the scale relates to actual dance experience and behavior) was assessed using dance experience measures. Construct validity (whether the scale measures the theoretical construct as expected) was evaluated through convergent and known-groups analyses. Convergent validity examined relationships with theoretically related constructs including mobility measures, self-rated general health, and arts engagement. Known-groups validity compared dance self-efficacy scores across groups expected to differ including active vs. insufficiently active, health conditions (1–2 vs. 3+), education levels, and cultural and/or linguistic diversity status. Higher dance self-efficacy scores were expected for participants who were younger, with more dance experience, better health and mobility, more active, higher engagement in arts and creative groups, higher education levels, and participants born in Australia or speaking English at home.

Different statistical methods were used to analyze relationships between dance self-efficacy scores and external variables based on their measurement characteristics. Linear regression was used for continuous variables, with separate models run for each predictor. Independent samples t-tests were used for two-group comparisons, and one-way ANOVAs for three or more group comparisons. Statistical assumptions were examined for all analyses. To control for multiple comparisons across 16 validity analyses, a Bonferroni correction was applied, setting the significance level at *p* < 0.003. Effect sizes were calculated for all analyses: R^2^ and unstandardized coefficients (b) for regression, Cohen’s d for *t*-tests, and eta-squared (η^2^) for ANOVAs, with effect size interpretations following Cohen’s conventions (small: *d* = 0.20, η^2^ = 0.01; medium: *d* = 0.50, η^2^ = 0.06; large: *d* = 0.80, η^2^ = 0.14).

#### 2.7.7 Identification of confidence indicators

Confidence indicator variables were identified as external benchmarks that correlate with scale scores to demonstrate validity and to differentiate between known groups. These additional items provide practitioners and researchers with additional measures to inform assessments of dance capability and can be used as standardized assessments of important factors that contribute to dance self-efficacy in older people.

## 3 Results

### 3.1 Item analysis

Item means (*M*) ranged from 2.71 to 3.96 (on the five-point scale), standard deviations (SD) ranged from 1.06 to 1.38. There was no missing data. All items showed the full response range and acceptable distributions without problematic floor or ceiling effects. No items met the threshold criteria for removal during initial screening.

Inter-item correlations showed no obvious issues. Item-rest correlations ranged from 0.611 to 0.885, with all items exceeding the minimum threshold. However, 11 items demonstrated correlations above 0.80, suggesting potential redundancy. Internal consistency for the 32-item scale was exceptionally high (Cronbach’s α = 0.98, McDonald’s ω = 0.98), indicating substantial item overlap that could limit factor differentiation (Boateng et al., 2018). Following content review, six items were strategically removed: two items with correlations above 0.85 and five additional items with correlations above 0.80 that were conceptually similar to retained items. This strategic removal reduced redundancy while preserving construct breadth, resulting in 25 items for factor analysis (α = 0.97, ω = 0.97).

### 3.2 Exploratory factor analysis

Two separate EFA models were tested using Sample 1 (*N* = 97). EFA of the six original dance self-efficacy items supported a unidimensional structure with excellent model fit [χ^2^(9) = 11.43, *p* = 0.25; CFI = 0.99, TLI = 0.99; RMSEA = 0.05, SRMR = 0.03] and high internal consistency (α = 0.90, ω = 0.90). Factor loadings ranged from 0.65 to 0.84, indicating that all items loaded substantially on a single factor representing general dance self-efficacy.

Exploratory Factor Analysis of the 25-item pool revealed a three-factor solution explaining 70.0% of total variance (eigenvalues: 16.26, 1.91, 1.50) but demonstrated poorer model fit [χ^2^(250) = 460.19, *p* < 0.001; CFI = 0.91, TLI = 0.88; RMSEA = 0.09, SRMR = 0.04]. The three extracted factors demonstrated theoretical coherence and high internal consistency: Factor 1 (dance task self-efficacy; α = 0.95) items related to performing movements, remembering steps, learning sequences, and trying different dance styles; Factor 2 (enjoyment-scheduling; α = 0.94) items covered enjoying group dancing and scheduling organized dance activities; and Factor 3 (barriers; α = 0.94) items addressed physical endurance and challenges such as dancing when tired or stressed. Factor loadings ranged from 0.48 to 0.89. Fit indices for all EFA and CFA models are displayed in Table 3.

**TABLE 3:** Fit indices for models assessed with EFA and CFA.

Model fit indices	McDonald’s ω ^3^	RMSEA^4^	CFI^5^	TLI^6^	SRMR^7^	χ^2^ (df)^8^	*P*χ^2^
**EFA^1^ (*N* = 97)**							
Model 1 (6 items)	0.90	0.05	0.99	0.99	0.03	11.43 (9)	0.25
Model 2 (25 items)	0.94	0.09	0.91	0.88	0.04	460.2 (250)	0.00
Model 3 (12 items)	0.95	0.10	0.96	0.94	0.03	86.7 (43)	0.00
**CFA^2^ (*N* = 192)**							
Model 4 (6 items)	0.88	0.11	0.96	0.94	0.04	31.5 (9)	0.00
Model 5 (12 items)	0.95	0.12	0.92	0.90	0.05	198.3 (53)	0.00

^1^EFA, exploratory factor analysis.

^2^CFA, confirmatory factor analysis.

^3^ω, McDonald’s omega.

^4^RMSEA, root mean square error of approximation.

^5^CFI, comparative fit index.

^6^TLI, Tucker-Lewis index.

^7^SRMR, standardized root mean square residual.

^8^χ^2^ (df), chi-square test (degrees of freedom); p, *p*-value.

### 3.3 Strategic item selection and scale construction

Following systematic procedures (Boateng et al., 2018), we retained the six original items and strategically added new items, prioritizing theoretical importance and construct coverage. Items were added incrementally (1–2 items per stage) to monitor factor structure and model fit changes. Two scheduling items were added first, followed by the highest-loading items from the three factors identified in the 25-item analysis.

As items from different domains were added, the unidimensional structure shifted toward a multifactorial solution. The dance task self-efficacy factor was retained, but the enjoyment-scheduling factor and the barriers to dancing factor merged. At 13 items, a stable 2-factor structure emerged with improved theoretical coverage and acceptable fit (Marsh et al., 2004). To balance the factors, the lowest-loading non-original item was removed, producing a final 12-item scale [χ^2^(43) = 86.72, *p* < 0.001; CFI = 0.96, TLI = 0.94; RMSEA = 0.10, SRMR = 0.03] with excellent reliability (α = 0.95, ω = 0.95). The 12-item model fit was poorer than the six-item model, but comparable to the 25-item solution, achieving gains in construct coverage while retaining acceptable psychometric properties.

### 3.4 Confirmatory factor analysis

Confirmatory factor analysis (CFA) was conducted on the independent validation sample (*N* = 192) to test the factor structures identified in EFA (Table 3). The six-item unidimensional model demonstrated acceptable fit [χ^2^(9) = 31.5, *p* < 0.001; CFI = 0.96, TLI = 0.94; RMSEA = 0.11, SRMR = 0.04], while the 12-item two-factor model showed comparable fit [χ^2^(53) = 198.3, *p* < 0.001; CFI = 0.92, TLI = 0.90; RMSEA = 0.12, SRMR = 0.05].

Standardized factor loadings are presented in Table 4 (six original items in bold). Some loadings exceeded 1.0, which can occur with highly correlated factors and strong item communalities, but did not indicate model misspecification given acceptable fit indices. The correlation between the two factors was strong (*r* = 0.80, *p* < 0.001), indicating substantial shared variance while maintaining discriminant validity. Both factors demonstrated excellent internal consistency: Factor 1 (Barriers and scheduling self-efficacy, α = 0.93, ω = 0.93) and Factor 2 (Dance task self-efficacy, α = 0.90, ω = 0.91). Overall scale reliability was excellent (α = 0.95, ω = 0.95).

**TABLE 4:** Standardized factor loadings for the 12-item scale using EFA and CFA.

Factor and items	EFA^3^	CFA^4^
**Factor 1: barriers and scheduling self-efficacy**	**1.0**	**1.0**
I can enjoy dancing even if I think I am not dancing very well	0.95	1.1
***I can enjoy dancing with a group^1^***	0.82	1.1
I can prioritize dance sessions when I make plans with friends and family and other appointments	0.79	1.2
I can arrange my schedule to include regular group dance sessions	0.60	1.0
I can do a group dance session when I am feeling stressed or depressed	0.55	0.85
***I can cope with the physical effort involved in dancing^1^***		
**Factor 2: dance task self-efficacy**	**0.98**	**1.0**
I can learn a complicated dance with many different steps	0.94	0.85
***I can remember the dance steps^1^***	0.64	0.98
***I can dance in time with the music^2^***	0.63	0.93
***I can follow instructions from the dance teacher or leader^2^***	0.54	1.0
I can try a style of dancing that is new to me	0.44	0.77
***I can dance with a partner I do not know^2^***	0.44	0.77

Items from the original 6-item dance self-efficacy scale displayed in bold and italic.

^1^Original dance self-efficacy item.

^2^Original dance self-efficacy item edited for clarity following expert review.

^3^EFA, exploratory factor analysis (standardized factor loadings reported).

^4^CFA, confirmatory factor analysis (standardized factor loadings reported).

### 3.5 Scale construction, scoring, and scale cutoffs

The final DanSES-60+ comprises 12 items organized into two subscales: Barriers and scheduling self-efficacy (six items) and Dance task self-efficacy (six items). Scale scores are calculated by summing item responses, with higher scores indicating greater dance self-efficacy. The total scale scores range from 12 to 60, subscale scores range from 6 to 30. Scale, subscale and item scores are presented in Table 5.

**TABLE 5:** DanSES-60+ scale, subscale and item scores.

Scale, subscale, items	*M* ^1^	Mdn^2^	SD^3^	Skew	Kurtosis	Min^4^	Max^5^	Item-rest^6^
DanSES-60+ total	42.14	43	11.12	−0.58	−0.26	12	60	–
Barriers and scheduling subscale	21.95	23	5.95	−0.69	−0.18	6	30	–
Enjoy poor performance	3.72	4	1.09	−0.69	−0.16	1	5	0.78
Enjoy group dancing	3.95	4	1.11	−0.94	0.10	1	5	0.83
Prioritize dance sessions	3.52	4	1.21	−0.54	−0.55	1	5	0.77
Arrange schedule	3.73	4	1.23	−0.76	−0.36	1	5	0.80
Dance stressed/depressed	3.39	4	1.17	−0.41	−0.68	1	5	0.77
Cope with physical effort	3.64	4	1.10	−0.56	−0.41	1	5	0.74
Dance task subscale	20.19	21	5.74	−0.32	−0.66	6	30	–
Learn complicated dance	2.71	3	1.26	0.26	−0.96	1	5	0.73
Remember steps	3.14	3	1.08	0.02	−0.72	1	5	0.71
Dance in time	3.87	4	1.17	−0.75	−0.46	1	5	0.74
Follow teacher	3.83	4	1.06	−0.64	−0.44	1	5	0.80
Try new dance style	3.37	4	1.21	−0.40	−0.76	1	5	0.79
Dance new partner	3.26	3	1.24	−0.25	−0.96	1	5	0.62

Dance self-efficacy final scale items are abbreviated.

^1^M, mean.

^2^Mdn, median.

^3^SD, standard deviation.

^4^Min, minimum value.

^5^Max, maximum value.

^6^Item-rest, item-rest correlation.

Individual items showed good distributional properties, all items used the full five-point response range. The lowest scoring items covered learning a complicated dance (*M* = 2.71) and remembering dance steps (*M* = 3.14). Participants recorded highest confidence for being able to enjoy dancing with a group (*M* = 3.95) and dancing in time to music (*M* = 3.87). Item-rest correlations ranged from 0.62 to 0.83, all exceeding the recommended minimum of 0.30 (Clark and Watson, 1995).

The DanSES-60+ showed good distributional properties (*M* = 42.14, Mdn = 43, SD = 11.12, range = 12–60). The distribution showed slight negative skew (−0.58) and normal kurtosis (−0.26), indicating that participants overall were reasonably confident about dancing with adequate score variability. Both subscales showed similar distributions with slight negative skew, with participants marginally more confident about coping with barriers and scheduling than performing dance tasks. Due to the negative-skewed distribution, tertile ranks were used to create scale cutoffs. Low dance self-efficacy was defined as scores less than 40 (33.3rd percentile = 39), moderate dance self-efficacy as scores 40–49, and high dance self-efficacy as scores of 50 and above (66.6th percentile = 48). Cutoff values were rounded to the nearest decade for ease of interpretation by practitioners.

### 3.6 Internal consistency and test-retest reliability

The DanSES-60+ total scale (α = 0.95, ω = 0.95), Barriers and scheduling subscale (α = 0.93, ω = 0.93) and Dance task subscale (α = 0.90, ω = 0.90) all demonstrated excellent internal consistency reliability. The scale also showed good temporal stability over a 2-week interval (*N* = 80; *r* = 0.85, *p* < 0.001; ICC = 0.84, 95% CI [0.77, 0.90]).

### 3.7 Criterion validity and confidence indicators

Relationships between DanSES-60+ scores and dance experience, age, gender, mobility, and falls history were examined to establish criterion and preliminary construct validity. Current dance participation and regular dancing over the past 5 years were both significantly related to dance self-efficacy scores. Current dancers had higher scores (*M* = 48.10, SD = 7.64) than people not dancing (*M* = 36.66, SD = 11.02; t(285) = 10.12, *p* < 0.001, *d* = 1.20). Participants who danced regularly over 5 years also scored higher (*M* = 47.74, SD = 8.18) than non-dancers (*M* = 35.74, SD = 10.62; t(285) = 10.73, *p* < 0.001, *d* = 1.27). Dance experience level was strongly related to total scores (R^2^ = 0.44, *b* = 8.08, 95% CI(b) [7.02, 9.14], β = 0.66, *t* = 15.06, *p* < 0.001), with each level increase corresponding to an eight-point increase in dance self-efficacy scores.

Neither age nor recent fall history were significantly related to dance self-efficacy scores. Age showed no relationship with total scores (R^2^ = 0.001, *b* = −0.04, 95% CI(b) [−0.22, 0.15], β = −0.02, *t* = 0.39, *p* = 0.69), and participants with and without recent falls did not differ significantly (No falls: *M* = 42.57, SD = 10.83; Falls: *M* = 40.06, SD = 12.23; t(280) = 0.11, *p* = 0.11). However, self-rated mobility was significantly related to dance self-efficacy scores [t(280) = 5.33, *p* < 0.001]. Participants with difficulty with climbing stairs and/or walking scored lower [*M* = 35.31, SD = 10.83] than those without difficulties [*M* = 42.71, SD = 10.54], with a four-point score difference on each subscale, a large effect (*d* = 0.79).

Although recent falls were not associated with dance self-efficacy scores, a rejected item (“I can dance on my own without holding on to anything and not lose my balance”) was significantly related to both recent falls [No falls: *M* = 4.07, SD = 1.11; Falls: 3.39, SD = 1.46; t(280) = 4.02, *p* < 0.001, *d* = 0.57] and total scale scores (R^2^ = 0.33, *b* = 5.22, 95% CI(b) [4.36, 6.08], β = 0.58, *t* = 11.96, *p* < 0.001), with one-point increase in balance confidence corresponding to a five-point increase in dance self-efficacy scores.

Three confidence indicators were identified for use alongside the DanSES-60+: Dance experience level item (GOLD-DSI; Rose et al., 2020), current dance participation (Merom et al., 2016a,2016b), and the dance balance confidence item. These indicators represent dance experience, current practice, and perceived stability while dancing respectively, and all demonstrated strong associations with dance self-efficacy total scores, providing evidence of excellent criterion validity. For the dance balance confidence item, 30% of participants reported poor confidence (scores ≤ 3), 27% reported moderate confidence (score of 4), and 43% reported high confidence (score of 5).

### 3.8 Construct validity

The DanSES-60+ demonstrated strong convergent and known-groups validity. Dance self-efficacy scores were significantly related to self-reported general health (R^2^ = 0.50, *b* = 5.58, 95% CI(b) [4.44, 6.73], β = 0.50, *t* = 9.60; *p* < 0.001). Although dance self-efficacy was unrelated to education level [F(2, 279) = 2.27, *p* = 0.11], it was significantly associated with overall participation in arts and creative groups (R^2^ = 0.13, *b* = 2.74, 95% CI(b) [1.92, 3.56], β = 0.37, *t* = 6.59; *p* < 0.001). Male respondents scored significantly lower in dance self-efficacy than female respondents [Male: *M* = 35.83, SD = 12.27; Female: *M* = 43.70, SD = 10.31; t(279) = 5.05, *p* < 0.001, *d* = 0.16].

Both activity levels and health conditions were related to dance self-efficacy scores. Respondents meeting physical activity guidelines had significantly higher DanSES-60+ scores than inactive respondents [Active: *M* = 43.48, SD = 10.34; Inactive: *M* = 39.03, SD = 12.26; t(280) = 3.20, *p* = 0.002, *d* = 0.40]. People with fewer health conditions also scored higher in dance self-efficacy [0–2 health conditions: *M* = 43.59, SD = 9.92; 3+ health conditions: *M* = 37.79, SD = 12.53; t(244) = 3.89, *p* < 0.001, *d* = 0.54]. There was no difference in DanSES-60+ scores between people born in or outside Australia [cultural diversity (31.1% respondents); t(280) = 0.83, *p* = 0.41], or between people who spoke English vs. another language at home [linguistic diversity (15.6% respondents); t(279) = 1.53, *p* = 0.13].

Table 6 displays the complete DanSES-60+, with the prompt, dance self-efficacy items, and confidence indicator questions. Supplementary File 1 provides the DanSES-60+ scale with scoring guide and brief administration instructions for researchers and practitioners.

**TABLE 6:** DanSES-60+ prompt, dance self-efficacy items, and confidence indicator questions.

DanSES-60+		Scale				
*Prompt: Whether or not you have danced before, how confident are you that you can do the following*		Not at all confident	A little confident	Moderately confident	Confident	Very confident
**Factor 1: barriers and scheduling self-efficacy**						
1.	I can enjoy dancing even if I think I am not dancing very well	1	2	3	4	5
2.	I can enjoy dancing with a group	1	2	3	4	5
3.	I can prioritize dance sessions when I make plans with friends and family and other appointments	1	2	3	4	5
4.	I can arrange my schedule to include regular group dance sessions	1	2	3	4	5
5.	I can do a group dance session when I am feeling stressed or depressed	1	2	3	4	5
6.	I can cope with the physical effort involved in dancing	1	2	3	4	5
**Factor 2: dance task self-efficacy**						
7.	I can learn a complicated dance with many different steps	1	2	3	4	5
8.	I can remember the dance steps	1	2	3	4	5
9.	I can dance in time with the music	1	2	3	4	5
10.	I can follow instructions from the dance teacher or leader	1	2	3	4	5
11.	I can try a style of dancing that is new to me	1	2	3	4	5
12.	I can dance with a partner I do not know	1	2	3	4	5
**Dance balance confidence item**						
13.	I can dance on my own without holding on to anything and not lose my balance	1	2	3	4	5
**Dance experience level item (GOLD-DSI)**		**None at all**	**Beginner**	**Intermediate**	**Advanced**	**Professional**
14.	I would classify my level of experience with dancing as	1	2	3	4	5
**Current dance participation**		**No**	**Yes**	**–**	**–**	**–**
15.	Are you currently attending group dance sessions or lessons (e.g., Ballroom, Zumba, Line dancing, Traditional Indian dance) once per week on most weeks?	0	1	–	–	–

## 4 Discussion

This study developed and validated the Dance Self-Efficacy Scale for older adults (DanSES-60+), extending the original six-item measure to provide a more comprehensive assessment of dance self-efficacy. The final 12-item scale comprises two related factors—“barriers and scheduling self-efficacy” and “dance task self-efficacy”—with acceptable fit. The scale demonstrated excellent reliability, temporal stability, and strong validity through associations with dance experience, mobility, physical activity/inactivity, health, gender, and participation in arts and creative groups. These associations remained significant after accounting for multiple comparisons. Age, falls history, education, and cultural and/or linguistic diversity showed no relationship with scores, indicating the potential for dance to be an inclusive activity for older people.

Although the original six-item scale performed well, the 12-item DanSES-60+ expands the barriers to dancing content to include self-efficacy for scheduling regular attendance at organized dance programs and attendance despite low mood, which are important for ongoing adherence. The dance task factor also represents a broader variety of dance activities that older adults have described as challenging (Waugh, 2022), with original items edited for greater clarity. The 12-item scale was deliberately designed to be brief, balancing comprehensive assessment with practical utility for both research and real-world applications where minimizing participant burden is often essential. The measure further incorporates a dance balance confidence item that serves as a valuable indicator related to falls history alongside standardized measures of dance experience that provide important context for understanding participants’ capabilities for dancing. The entire 15-item assessment has been tested on a representative of older people from the general population and is more useful for both practice and research. Given these substantial improvements, the DanSES-60+ is designed to completely replace the older six-item scale as the gold-standard measure of dance self-efficacy in people aged 60 years and over.

### 4.1 Psychometric performance and theoretical implications

The two-factor structure partially aligned with theoretical expectations established in exercise self-efficacy research (Maddux, 1995; Rodgers et al., 2008). Although three self-efficacy domains were proposed initially, scheduling items loaded with barrier items rather than forming a distinct factor. The results map onto Maddux’s (1995) task and coping with barriers and scheduling self-efficacy conceptualization. The dance task factor captured cognitive-motor, and social demands of the activity itself, while the barriers and scheduling factor addressed broader challenges (enjoying group activities, performance anxiety, emotional coping, scheduling and prioritizing organized dance activities). This structure suggests general exercise self-efficacy scales may inadequately represent complex activities involving cognitive-motor, social, and enjoyment dimensions beyond basic motor tasks (e.g., walking). The strong inter-factor correlation indicates that the two self-efficacy domains are closely related in practice.

The DanSES-60+ demonstrated good reliability and temporal stability, indicating that dance self-efficacy represents a relatively stable characteristic (Bandura, 2006). The scale showed robust validity evidence across multiple domains and appropriately distinguished individuals based on dance involvement. The positive associations with health, physical activity, and mobility replicated results from the original six-item scale (Waugh et al., 2023) and self-efficacy research (McAuley et al., 2011b). The relationship with broader engagement in arts and creative groups extends validity evidence, suggesting dance self-efficacy may reflect confidence for creative activities. Unlike the original six-item scale (Waugh et al., 2023), men scored lower in dance self-efficacy, likely because they had less dance experience overall; this reflects broader population patterns where women dance more frequently than men (Merom et al., 2012; Waugh et al., 2024), whereas men in the original validation had self-selected into a dance program.

Age and falls history were not associated with dance self-efficacy scores, contrasting the typical age-related declines in exercise self-efficacy (Anderson-Bill et al., 2011), and established relationships between falls and both falls self-efficacy and exercise self-efficacy (McAuley et al., 2011b; Li et al., 2023). This divergence may reflect dance’s unique combination of motor skill, artistic expression, and social interaction providing alternative mastery sources that compensate for declining capabilities. The findings suggest that older adults with falls history may not be deterred from dance programs, potentially breaking the cycle of falls leading to exercise avoidance. Unlike exercise self-efficacy (McAuley et al., 2011b), dance self-efficacy appears to be evaluated against age-appropriate capability expectations rather than absolute standards.

Relationships between dance self-efficacy, education, and cultural and/or linguistic diversity were absent, despite typical associations between education and arts participation (Bone et al., 2021) and cultural and linguistic barriers to social and physical activities among older adults (Georgeou et al., 2021). However, Bone et al. (2021) found education was unrelated to active arts participation in the United States, suggesting participatory arts differ from arts event attendance, which is more socioeconomically determined. Dance may be acceptable across diverse older adult populations (Fancourt et al., 2020; Golden et al., 2023), important given exercise participation is typically education and socioeconomically dependent (Harris et al., 2024), suggesting dance may reduce participation barriers.

### 4.2 Applications in research and practice

The DanSES-60+ provides researchers and practitioners with a validated tool to advance understanding of dance for health.

#### 4.2.1 Research applications

For program evaluation, the DanSES-60+ enables standardized pre-post and progressive assessment to determine intervention effectiveness (McAuley et al., 2011b). We recommend assessing dance self-efficacy at multiple time points (e.g., every 4–6 weeks) to understand change over time and relative to attendance, particularly for longer programs. High-performing programs should improve self-efficacy, particularly among beginners (McAuley et al., 2011b). However, novices may over-estimate self-efficacy for unfamiliar activities (McAuley et al., 2011a), which should be considered when interpreting baseline scores and early changes.

The scale facilitates standardized assessment across programs, enabling effectiveness comparisons across different dance styles and programs (Picorelli et al., 2014; Beck et al., 2016; Waugh et al., 2024) and programs (Picorelli et al., 2014; Beck et al., 2016), and including additional design features such as. Researchers can evaluate how specific program features impact dance self-efficacy such as structured “at home” practice materials, performance opportunities, and facilitation approaches (Fortin, 2018; Waugh et al., 2024). Theory-informed behavioral change strategies to support adherence and engagement should improve activity-specific self-efficacy (Chase, 2015; Carey et al., 2019; Gilchrist et al., 2024). The two-factor DanSES-60+ structure enables further examination of whether task and barrier/scheduling self-efficacy differentially predict outcomes (e.g., functioning vs. wellbeing) and respond differently to specific intervention components.

The DanSES-60+ may help explain inconsistent findings across dance trials by better accounting for individual differences and exploring how individual characteristics interact with program features (Picorelli et al., 2014; Beck et al., 2016; Waugh et al., 2024, 2025). Researchers can use the scale to examine moderation effects (do programs work differently for people with different baseline dance self-efficacy), mediation effects (do changes in dance self-efficacy explain health outcomes), differential attrition (do people with lower dance self-efficacy drop out more), and dose-response relationships (how attendance relates to dance self-efficacy and outcomes). For example, in our falls prevention trial (Merom et al., 2016b), stronger program attendance was associated with improved physical function only for people with low dance self-efficacy at baseline (Waugh et al., 2025).

Research using the six-item scale demonstrated dance self-efficacy mediated relationships between program attendance and physical functional outcomes, indicating improvements in health outcomes may be explained by increased dance self-efficacy (Waugh et al., 2025). The findings suggest dance self-efficacy may reflect not only participation frequency (attendance), but engagement quality. Individuals with stronger self-efficacy may dance with greater effort, take more risks, perform more expansive movements, and engage more fully, improving physical function and possibly translating to motor changes beyond dance sessions. We termed this the “Bananarama Confidence-Performance Pathway,” emphasizing that for dance and potentially other arts-based approaches, “it’s not (only) what you do, it’s the way that you do it, that gets results” (Waugh et al., 2025).

Dance combines multiple evidence-based “active ingredients” including mind-body exercise, motor learning, creative and artistic engagement within a group context, physical expression, music, and balance challenges (McCrary et al., 2021; Warren et al., 2022; Waugh et al., 2024). These components likely impact a range of health outcomes individually and interactively. Dance self-efficacy may capture holistic engagement across components of group dancing in ways that single functional outcome measures cannot, particularly for older people with chronic and neurodegenerative health conditions who may benefit from these integrated therapeutic elements and were represented in this community sample. Improved dance self-efficacy following intervention can therefore be interpreted as a meaningful outcome in itself, reflecting increased confidence and capability for sustained engagement in dance and related activities.

Finally, the three confidence indicator items enable screening participants by dance experience level (e.g., identifying beginner dancers) and dance balance confidence (e.g., identifying people who are unsure about maintaining their stability while dancing). Researchers can control for baseline dance experience and dance balance confidence in analyses. The dance balance confidence item can be compared with objective measures of balance and falls risk to examine relationships. The standardized dance experience measures enable consistent reporting across studies and examination of relationships between dance experience and other factors.

#### 4.2.2 Practice applications

Practitioners can use the DanSES-60+ for program evaluation to monitor whether programs maintain or improve dance self-efficacy over time. For new programs, new teachers, or new participants, lack of improvement or decline may indicate programs require modification in challenge level, instructional approach, or individualized support. For long-running programs with experienced participants, maintaining self-efficacy levels may be appropriate.

For participant screening, the scale cutoffs provide practical thresholds (low < 40, moderate 40–49, high ≥ 50) identify individuals with low dance self-efficacy who may require additional support to participate successfully, and individuals with high dance self-efficacy who may appreciate additional challenges to maintain engagement. Practitioners can monitor individual progress over time and assess whether support strategies are effective.

For streaming participants into different class levels and program matching, the scale cutoffs can be used to match participants to appropriately challenging program levels or types based on their dance self-efficacy, which indicates perceived capability for group dance. Healthcare providers involved in social prescribing can use these cutoffs to match older adults to programs suited to their current capabilities.

The dance balance confidence item (scored 1–5) indicates confidence for balance challenges while dancing, and potential falls risk. Scores of 3 or less indicate poor dance balance confidence and may suggest participants need additional support or modified balance challenges. The dance experience items indicate how much dance training or participation individuals have had previously.

### 4.3 Limitations and future directions

Several limitations should be considered. Although respondents were broadly representative of Australian older adults in cultural and/or linguistic diversity (Australian Bureau of Statistics, 2022), our recruitment strategy and English-language online survey administration likely excluded people with limited English proficiency who experience the greatest activity participation barriers. Respondents were more highly educated than the general population (Australian Institute of Health and Welfare., 2023), which may limit generalizability. The overrepresentation of women may limit applicability to men.

The cross-sectional design prevented examination of responsiveness of the DanSES-60+ to change over time, and experimental studies should establish intervention effects. Additionally, many dance programs for older people are designed for clinical groups, particularly people with neurodegenerative disorders such as Parkinson’s disease, dementia, or stroke which may affect self-efficacy for dancing periodically or progressively. Establishing appropriate use and interpretation guidelines for these contexts is essential. Further validation with more diverse populations and in different cultural contexts would strengthen the scale’s cross-cultural applicability.

Future research should also examine relationships with related constructs such as exercise self-efficacy and motivation to provide a comprehensive understanding of psychological processes underlying dance participation. Finally, researchers can further test the “Bananarama Confidence-Performance Pathway” hypothesis by examining whether dance self-efficacy and changes in dance self-efficacy reflect actual movement quality.

### 4.4 Conclusion

The DanSES-60+ provides the first validated tool to measure dance self-efficacy in older adults. The scale reflects the unique social, psychological, and practical challenges older adults face in community dance programs, and potentially other multifaceted group activities. The DanSES-60 + enables identification of participant needs and investigation of psychological mechanisms underlying program effects. The study addresses a measurement gap to enhance understanding of individual factors affecting dance program adherence, dance behavior, and health outcomes. Dance research and community programs can now incorporate standardized dance self-efficacy assessment to advance research and practice in supporting older adults’ engagement and health outcomes in dance activities.

## Data Availability

The datasets presented in this study can be found in online repositories. The names of the repository/repositories and accession number(s) can be found below: the datasets generated and analysed for this study can be found in the the Western Sydney University Research Direct repository and can be accessed via this link: https://doi.org/10.26183/nbvc-z832.
