# Supplementary material for: So you think you can dance? Development and validation of the Dance Self-Efficacy Scale for older adults (DanSES-60+) for research and practice
Source: Front Psychol. 2026 Jan 7;16:1712057. doi: 10.3389/fpsyg.2025.1712057 (PMC12819324; doi:10.3389/fpsyg.2025.1712057)
Supplement: Supplementary file 1 [file Data_Sheet_1.pdf]

# Dance self-efficacy scale for older adults (DanSES-60+): Items and scoring guide

**Overview**: Dance self-efficacy—dance confidence—is a person's beliefs in their capabilities to participate in organised group dance. The DanSES-60+ measures dance self-efficacy in people aged 60+ living in the community, with and without prior dance experience. An additional 3 items assess dance experience and dance balance confidence. The DanSES-60+ can be used to screen and stream participants into dance programs with different levels and identify support needs, monitor participant progress, and for dance program evaluation and research. For more information visit www.dancebrainhub.com

| **DanSES-60+ prompt and items** | | | **Scale** | | |
|---|---|---|---|---|---|
| *Prompt: Whether or not you have danced before, how confident are you that you can do the following:* | **Not at all confident** | **A little confident** | **Moderately confident** | **Confident** | **Very confident** |
| **FACTOR 1: BARRIERS AND SCHEDULING SELF-EFFICACY** | | | | | |
| 1. I can enjoy dancing even if I think I am not dancing very well | 1 | 2 | 3 | 4 | 5 |
| 2. I can enjoy dancing with a group | 1 | 2 | 3 | 4 | 5 |
| 3. I can prioritise dance sessions when I make plans with friends and family and other appointments | 1 | 2 | 3 | 4 | 5 |
| 4. I can arrange my schedule to include regular group dance sessions | 1 | 2 | 3 | 4 | 5 |
| 5. I can do a group dance session when I am feeling stressed or depressed | 1 | 2 | 3 | 4 | 5 |
| 6. I can cope with the physical effort involved in dancing | 1 | 2 | 3 | 4 | 5 |
| **FACTOR 2: DANCE TASK SELF-EFFICACY** | | | | | |
| 7. I can learn a complicated dance with many different steps | 1 | 2 | 3 | 4 | 5 |
| 8. I can remember the dance steps | 1 | 2 | 3 | 4 | 5 |
| 9. I can dance in time with the music | 1 | 2 | 3 | 4 | 5 |
| 10. I can follow instructions from the dance teacher or leader | 1 | 2 | 3 | 4 | 5 |
| 11. I can try a style of dancing that is new to me | 1 | 2 | 3 | 4 | 5 |
| 12. I can dance with a partner I do not know | 1 | 2 | 3 | 4 | 5 |

| Additional ‘indicator’ items (scored separately) | Scale | | | | |
|---|---|---|---|---|---|
| | **Not at all confident** | **A little confident** | **Moderately confident** | **Confident** | **Very confident** |
| **DANCE BALANCE CONFIDENCE** | | | | | |
| 13. I can dance on my own without holding on to anything and not lose my balance | 1 | 2 | 3 | 4 | 5 |
| **DANCE EXPERIENCE LEVEL** | **None at all** | **Beginner** | **Intermediate** | **Advanced** | **Professional** |
| 14. I would classify my level of experience with dancing as: | 1 | 2 | 3 | 4 | 5 |
| **CURRENT DANCE PARTICIPATION** | **No** | **Yes** | - | - | - |
| 15. Are you currently attending group dance sessions or lessons (e.g., Ballroom, Zumba, Line dancing, Traditional Indian dance) once per week on most weeks? | 0 | 1 | - | - | - |

## Scoring information

**Administration guide**: Time to complete is approximately 3-5mins.
**DanSES-60+ scoring**: Sum items 1-12 for a total dance self-efficacy score out of 60. Items 13-15 provide supplementary information for research and assessment but are not included in the total DanSES-60+ score.
**Subscale scoring**: ‘Barriers and scheduling self-efficacy’ items measure confidence in overcoming challenges to participation and maintaining regular attendance at organised group dance activities (items 1-6, range 6-30); ‘Dance Task Self-Efficacy’ measures confidence in performing dance activities and learning movements (items 7-12, range 6-30).
**Scale cutoffs**: Scores of less that 40 (<40) indicate *low dance self-efficacy*; Scores of 40-49 indicate *moderate dance self-efficacy*; Scores of 50 and over (≥50) indicate *high dance self-efficacy*. For the dance balance confidence item, scores of 3 or less indicate poor dance balance confidence and potentially higher falls risk.
**Psychometric properties**: People with higher DanSES-60+ scores tend to have more dance experience, better mobility and general health, be more physically active, and participate more in other arts and creative activities.
